# The Involvement of Aquaporin-4 in the Interstitial Fluid Drainage Impairment Following Subarachnoid Hemorrhage

**DOI:** 10.3389/fnagi.2020.611494

**Published:** 2021-01-26

**Authors:** E. Liu, Xianlong Peng, Haowen Ma, Yan Zhang, Xiaomei Yang, Yixuan Zhang, Linlin Sun, Junhao Yan

**Affiliations:** ^1^Department of Anatomy and Histology, School of Basic Medical Sciences, Peking University, Beijing, China; ^2^Department of Anatomy, School of Medicine, Shandong University, Jinan, China; ^3^Beijing Key Lab of Magnetic Resonance Imaging Technology, Peking University Third Hospital, Beijing, China

**Keywords:** aquaporin-4, interstitial fluid, subarachnoid hemorrhage, glymphatic system, rat

## Abstract

The mechanism of brain injury following subarachnoid hemorrhage (SAH) has not yet been clarified. The glymphatic system (GS), a glia-dependent waste clearance pathway, drains away soluble waste proteins and metabolic products, even some toxic factors from the brain. Aquaporin-4 (Aqp4) is highly expressed on the astrocyte foot processes and facilitates the interstitial fluid (ISF) transportation in the GS system. In this study, the role of Aqp4 in the GS injury after SAH was explored using Aqp4 gene knockout (Aqp4^−/−^) Sprague Dawley rats. The results of MRI, fluorescent imaging, and transmission electron microscopy (TEM) indicated that, after SAH, the inflow of cerebrospinal fluid (CSF) into the brain and the clearance of ISF from the brain were both significantly decreased. Meanwhile, the expression level of Aqp4 around the artery was markedly higher than that around the vein following SAH. Aqp4 knockout exacerbated the GS damage after SAH. In summary, after SAH, there was an apparent GS impairment, and Aqp4 played key roles in modulating the function of GS in the brain.

## Introduction

Subarachnoid hemorrhage (SAH) is mainly caused by the rupture of intracranial aneurysms, and the fatality rate is about 40% (Macdonald et al., 2007). Early brain injury (EBI) occurs within 72 h following SAH and accounts for about 60% of the deaths. The potential mechanisms of EBI include increased intracranial pressure, hypoxia, and ischemic insult in the whole brain, cerebral edema, blood-brain barrier (BBB) disruption, inflammation activation, and neuronal apoptosis (Gupta et al., 2013; Huang et al., 2013; Sabri et al., 2013; Wong et al., 2014). It is reported that EBI is a determining factor for the poor outcomes after SAH (Sehba et al., 2012). Therefore, how to effectively treat against EBI has become a major goal for patients with SAH. The previous studies about SAH were mainly focused on neuronal apoptosis, BBB disruption, inflammation, and oxidative stress (Gleichman and Carmichael, 2014; Sehba, 2015); however, until now, no significant breakthrough has been achieved. These results imply that there might be some other underlying mechanisms after SAH that play their key roles in the development of EBI and even in the outcome of SAH.

The glymphatic system (GS) drains the cerebrospinal fluid (CSF) into the brain interstitial space (ISS) through the para-arterial space (PAS), which is bound by the blood vessel and the astrocyte endfeet (Lei et al., 2017). Aquaporin-4 (Aqp4) is a member of the water channel family and is mainly distributed on the astrocyte endfeet. It facilitates CSF to enter into the brain parenchyma through the PAS and then mix with interstitial fluid (ISF) within ISS (Teng et al., 2018). The ISF carrying various waste proteins and metabolic products is cleared out of the brain through the paravenous space (PVS; Iliff et al., 2013). Our previous study showed that Aqp4 knockout aggravated the loss of neurons in the hippocampus and the BBB disruption after SAH (Liu et al., 2020). However, the potential injury mechanism and relationship with the GS are yet to be clarified.

There is experimental evidence that both GS and paravascular pathways are impaired after SAH (Luo et al., 2016; Pu et al., 2019; Liu et al., 2020). This implies that potentially harmful compounds released from extravasated blood cells will not be effectively cleared out from the ISF which may aggravate EBI and result in a dysfunction of the neurovascular unit.

In this study, by using the MRI technique and the high-resolution laser confocal microscopy methods, the alteration of CSF inflow into the brain and ISF clearance from the brain after SAH was assessed, along with a neurological scoring. Moreover, since Aqp4 constitutes an essential element of the GS, the study was performed in normal rats and in Aqp4 knockouts.

## Materials and Methods

This study was performed according to the national guidelines for the use of experimental animals, and the study protocols have been approved by the Ethics Committee of Peking University Health Science Center.

### Animals

The male Sprague Dawley rats and Aqp4^−/−^ rats (280–300 g) were divided into sham group, Aqp4^−/−^-sham group, SAH group, and Aqp4^−/−^-SAH group, respectively (*n* = 40 each group). The animals were housed under 12 h light/dark cycles. The temperature (22 ± 1°C) and humidity (60 ± 5%) in the room were controlled.

The transcription activator-like effector nuclease (TALEN)-mediated knockout approach was applied to generate Aqp4-deficient rats. Briefly, we designed and synthesized highly active TALENs against the following sequences: (5′-CACAGCAGAGTTCCTGG-3′) for the sense strand and (5′-GGATCCCACGCTGAGCA-3′) for the antisense strand. The messenger RNAs (mRNAs) of the TALENs were injected into the cytoplasm of pronuclear stage embryos to produce mutant founders (F0). F0, which lacked three base pairs, was crossed with the wild-type rat to produce the F1 generation. The heterozygous offspring of F1 were crossed to generate F2. The genomic analysis, performed by sequencing PCR products, showed that the pups were heterozygous. The Aqp4^−/−^ rats were viable and fertile and did not exhibit any gross abnormalities, which indicated that the TALEN-mediated Aqp4 mutation was stably inherited. The effects of Aqp4 mutation were confirmed using the Western blot.

### Establishment of SAH Model

The rat SAH model was established as reported by others (Bederson et al., 1995; Xiong et al., 2020). The rats were anesthetized *via* intraperitoneal injection of sodium pentobarbital (50 mg/kg) and maintained with 30 mg/kg/h sodium pentobarbital during the operation. A midline skin incision was performed to expose the right common carotid artery. The external carotid artery (ECA) and its branches were isolated and coagulated. Thereafter, a 3–0 nylon monofilament with a blunt tip was introduced into the right internal carotid artery (ICA) through the ECA stump to the anterior cerebral artery (ACA) near the anterior communicating artery, where the resistance was encountered. The suture was advanced 3 mm further to perforate the artery and then withdrawn. The sham animals underwent the same surgical operation except the suture was immediately withdrawn while the resistance was felt. The body temperature of the animal was maintained at 37.5°C. After the surgery, the rats were housed in heated cages until recovery.

### Neurological Function Evaluation

The neurological function was assessed at 24 h following SAH based on a previous study (Yang et al., 2019). This sensory-motor assessment system consists of six items, with scores from 0 to 3 or from 1 to 3 (maximum score = 18), such as spontaneous activity, symmetry of the limb movement, forepaw outstretching, climbing, body proprioception, and whisker stimulation response.

### Brain Water Content Assessment

The brain water content of each group was evaluated as previously reported (Yang et al., 2020). The rats were anesthetized by intraperitoneal injection of pentobarbital sodium (50 mg/kg; Abbott Laboratory, North Chicago, IL, USA). At 24 h after SAH, the brain was removed and weighed immediately (wet weight) and weighed again following drying in an oven at 105°C for 24 h (dry weight). The brain water content was calculated as follows: [(wet weight-dry weight)/wet weight] × 100%.

### Interstitial Fluid Tracking Technique

In this study, we used Gadolinium-diethylenetriaminepentaacetic acid (Gd-DTPA; Magnevist; Bayer Schering Pharma AG, Germany) under MRI or fluorescence dyes using confocal microscopy as tracers for ISF drainage. Firstly, we observed the inflow of CSF with Gd-DTPA or fluorescence dyes into the brain parenchyma using the cisterna magna injection method. Additionally, the hippocampus injection method was applied to reveal the ISF clearance from the brain.

### Injections of Gd-DTPA Tracer and MRI Scan

It is reported that Gd-DTPA is mainly distributed within the extracellular space and cannot permeate into the neurocytes and can be used as a good indicator for observing the ISF flow (Teng et al., 2018; Wang et al., 2019). Therefore, in this study, Gd-DTPA and MRI scan methods were used to detect the ISF drainage characteristic. Briefly, Gd-DTPA was diluted to 10 mmol/L with 154 mmol/L NaCl solution and then injected into the cisterna magna immediately after SAH to observe its influx into the brain at 0.5, 1, 2, and 3 h post injection. The anesthetized rat was placed prone on a stereotaxic instrument (Lab Standard Stereotaxic-Single, Stoelting Co, Illinois, USA) and secured using a head adaptor. A sagittal incision of the skin was made inferior to the occiput; the subcutaneous tissue and rectus capitis dorsalis major muscles were bluntly separated to expose atlanto-occipital membrane, which was incised to show the dura mater of the cisterna magna. The position of the animal was then adjusted so that the head formed a 135° angle with the body. Then, the dura mater was pierced with the injection pipette lateral to the arteria dorsalis spinalis, and 50 μl Gd-DTPA was injected into the cisterna magna over 5 min at a rate of 10 μl/min using a 50 μl microsyringe (Hamilton, Bonaduz AG, Switzerland). The rat was immediately placed in the scanner in a prone position to visualize tracer inflow from the cisterna magna into the hippocampus.

The Gd-DTPA injection into the hippocampus was also performed immediately after SAH to explore its clearance from the brain at 0.5, 1, 2, and 3 h post injection. The rat was immobilized in the stereotactic coordinate system, and a small trephine hole was made according to the stereotactic coordinates of the hippocampus (bregma: −5.0 mm, lateral: 5.0 mm, vertical: 6.0 mm). About 2 μl Gd-DTPA (10 mmol/L) was injected into the hippocampus using a 10 μl microsyringe (Hamilton, Bonaduz AG, Switzerland) at a rate of 0.2 μl/min through an automated drug administration system (Harvard Apparatus, USA). The rat was then quickly placed in the scanner to visualize the tracer clearance in the hippocampus.

A 3.0 T MRI system (Magnetom Trio, Siemens Medical Solutions, Erlangen, Germany) with an eight-channel wrist coil was applied to obtain the brain images of animals by running a T1-weighted magnetization-prepared rapid-acquisition with gradient echo (MP-RAGE) sequence. The acquisition parameters were as follows: echo time = 3.7 ms, repetition time = 1,500 ms, flip angle = 12°, inversion time = 900 ms, field of view = 267 mm, voxel = 0.5 mm^3^, matrix = 512 × 512, number of averages = 2, and phase-encoding steps = 96. The acquisition time for each rat was 290 s.

We used ImageJ software (National Institutes of Health, Bethesda, MD) to analyze the concentration of Gd-DTPA in the brain parenchyma at 0.5, 1, 2, and 3 h post injection and applied the software developed by our laboratory to calculate the diffusion rate, D*; the clearance rate, k′; and the half-life, t_1/2_, of Gd-DTPA in the hippocampus.

### Fluorescence Imaging

In this study, the fluorescence tracers were also applied to explore the ISF flow in the brain, owing to their impermeability to the cellular membrane and preservation within interstitial space as reported by others (Wang et al., 2019). The fluorescence tracers in this study, such as ALEXA-594 hydrazide (A594, 759D), Cascade Blue (CB, 10KD), FITC-dextran 2000 (FITC-d2000,2000KD), FITC(389D), and Texas Red (TR, 3kD) were constituted in the artificial CSF at a concentration of 0.05% (all from Invitrogen, Oregon, USA).

The fluorescence commixture of A594, CB, and FITC-d2000 (50 μl) was injected into the cisterna magna immediately after SAH at a rate of 5 μl/min to observe its influx into the brain. At 0.5 h after injection, the animal was perfused transcardially with 300 ml physiological saline within 15 min followed by 300 ml 4% paraformaldehyde. The brain was removed and post-fixed in 4% paraformaldehyde (6 h, 4 C). The slices of the hippocampus (coronary position, 2 μm) were prepared to observe the entry of the fluorescence commixture from the CSF into the hippocampus using laser scanning confocal microscopy (LSCM) SP8 (Leica, Germany).

Additionally, to observe the ISF clearance in the hippocampus, the fluorescence commixture of A594, CB, and FITC-d2000 (2 μl) was also injected into the hippocampus immediately after SAH at a rate of 0.2 μl/min. Thirty minutes after injection, the rat was perfused transcardially with 300 ml physiological saline for 15 min followed by 300 ml 4% paraformaldehyde. The rat brains were removed and post-fixed in 4% paraformaldehyde (6 h, 4°C). The brain slices of the hippocampus (coronary position, 2 μm) were prepared for LSCM SP8 examination to observe the distribution of the fluorescence commixture in the hippocampus.

### Transmission Electron Microscope Examination

The anesthetized rats were injected with 2 μl of 10 nm gold nanoparticles (600KD, QDSphere, USA) into the right hippocampus immediately after SAH at a rate of 0.2 μl/min. Injection pipettes were left *in situ* for 5 min to prevent the reflux. The rats were sacrificed and perfusion-fixed at 3 h after injection. The hippocampal tissue block (<1 mm^3^) adjacent to the injection site was prepared for TEM examination to observe the distribution of gold nanoparticles.

### Immunofluorescence Staining

At 24 h after SAH, as reported by others (Cai et al., 2020), the hippocampus sections were incubated overnight with anti-α smooth muscle actin (1:300, Abcam, Cambridge, USA), anti-laminin (1:200, Abcam, Cambridge, USA), and anti-Aqp4 (1:500, Abcam, Cambridge, USA) and developed with corresponding secondary antibodies (Abcam, Cambridge, USA). The photomicrographs of immunofluorescence staining were captured using the LSCM SP8.

### Western Blot

At 24 h after SAH, the protein sample was prepared by homogenizing the hippocampus with RIPA lysis buffer (Santa Cruz Inc., CA, USA). The protein concentration was measured using a detergent compatible assay (Bio-Rad, DC protein assay). The protein samples (45 μg) were loaded on an SDS-PAGE gel, electrophoresed, and transferred onto a nitrocellulose membrane. The membrane was incubated with primary antibodies at 4°C overnight and then incubated with the corresponding secondary antibodies for 1 h at room temperature. The immunoblots were probed by applying the ECL Plus Chemiluminescence Reagent Kit (Amersham Biosciences, Arlington Heights, IL). The results were then analyzed using ImageJ software (National Institutes of Health, Bethesda, MD, USA; Zhang et al., 2020).

### Statistics

The data in this study were expressed as mean ± SD. Statistical analyses were performed with SPSS 19.0 software (SPSS Inc., Chicago, IL, USA). The one-way analysis (ANOVA) and the Tukey's multiple comparison test were used for comparison among the groups. A value of *p* < 0.05 was considered statistically significant.

## Results

### Mortality

In this study, no animal died in both sham and Aqp4^−/−^-sham groups, and the mortalities of rats in the SAH group and the Aqp4^−/−^-SAH group were 32.5% (13/40) and 37.5% (15/40), respectively. The success rate of SAH induction in this study was 76.2% (80/105).

### Neurological Function and Brain Edema Evaluation

**Figure 1:**
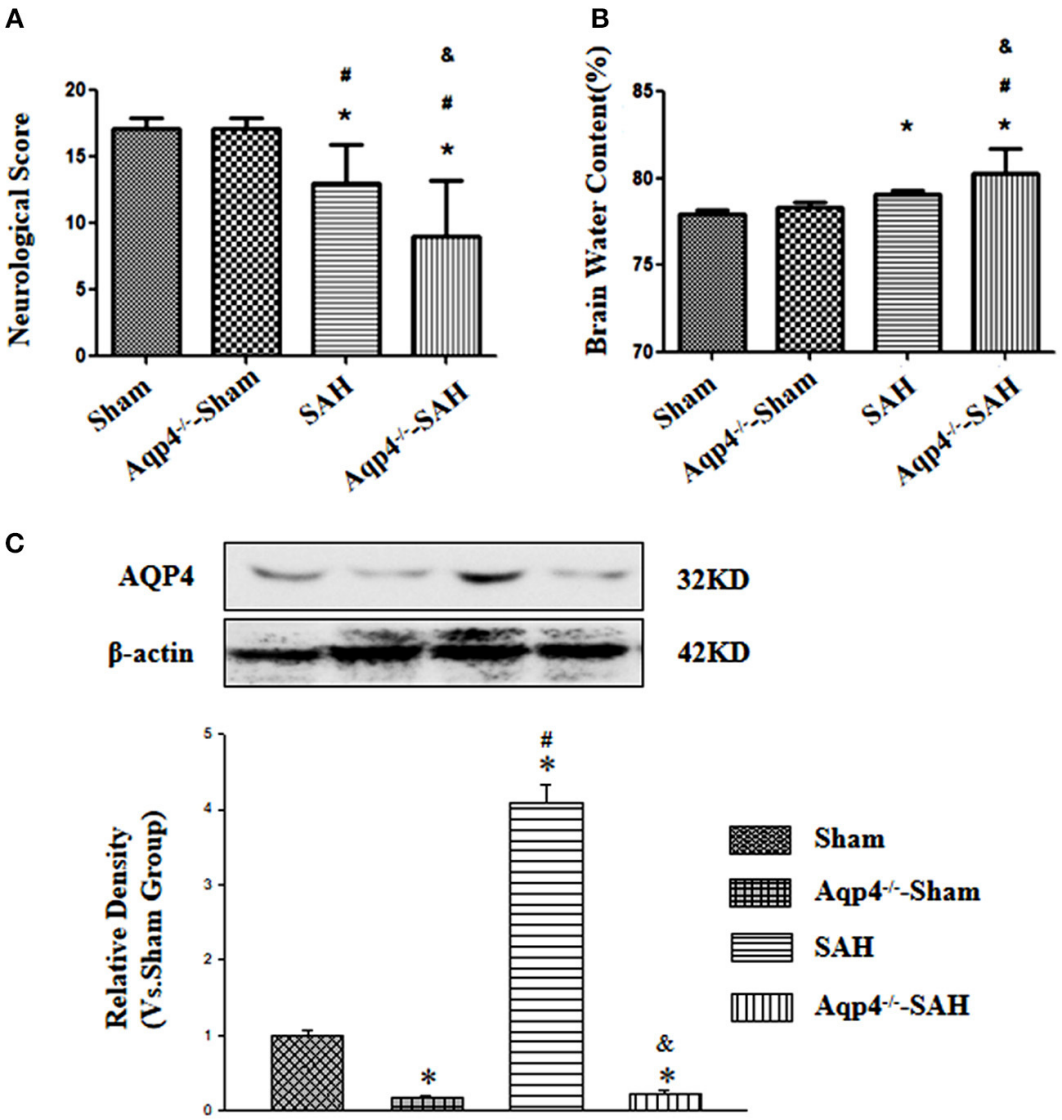
The effects of Aqp4 knockout on the neurological function and brain edema after SAH. There are evidently neurological deficits and brain edema after SAH, which can be aggravated by Aqp4 knockout **(A,B)**. The expression of Aqp4 in the hippocampus is significantly increased after SAH; additionally, the expression level of Aqp4 in the hippocampus is markedly decreased in the Aqp4-knockout rats **(C)**. *p < 0.05 vs. the sham group, #*p* < 0.05 vs. the Aqp4^−/−^-sham group, ^&^*p* < 0.05 vs. the SAH group.

Aquaporin-4 knockout had little influence on the neurological function and the brain water content (*p* > 0.05). After SAH, there were evidently neurological deficits and brain edema, especially in the animals of the Aqp4 knockout group (*p* < 0.05; Figures 1A,B)

In addition, we had also confirmed the efficiency of Aqp4 knockout using the Western blot. The results indicated that the expression level of Aqp4 in the hippocampus was markedly decreased in the Aqp4^−/−^-sham and Aqp4^−/−^-SAH groups, and interestingly, the expression level of Aqp4 was significantly increased in the SAH group (*p* < 0.05, Figure 1C).

### Injections of the Gd-DTPA Tracer

**Figure 2:**
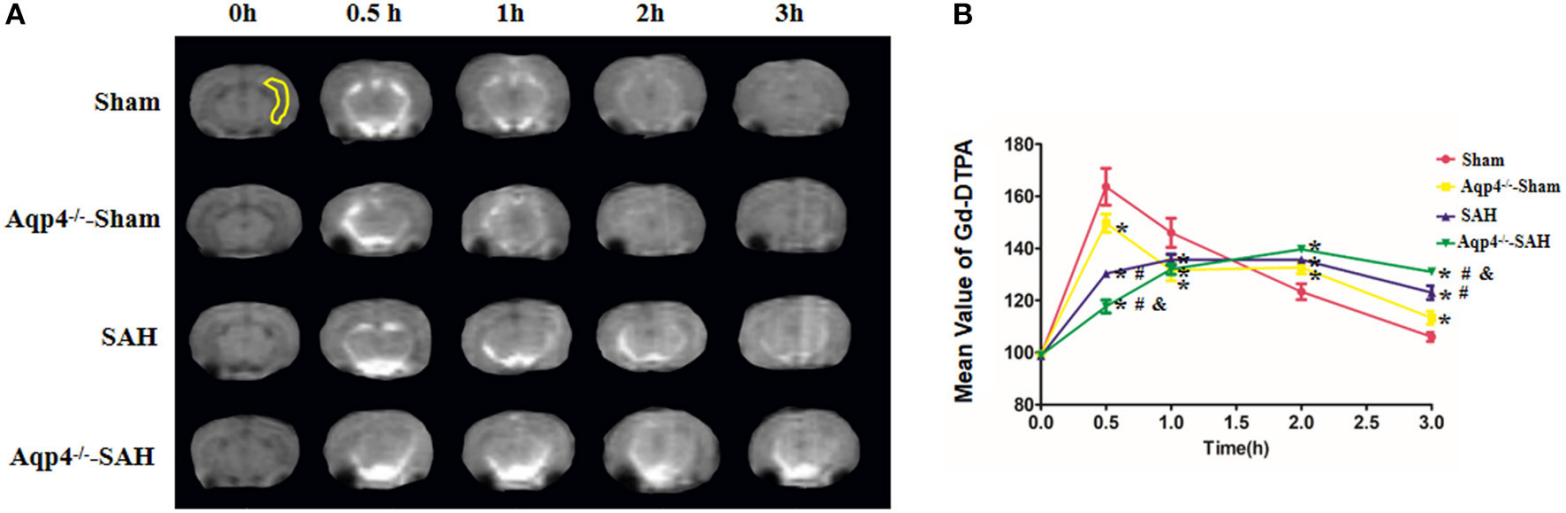
Distribution of Gd-DTPA injected into the cisterna magna. In the sham group, Gd-DTPA is widely distributed in the subarachnoid space and ventricles and gradually flows into the hippocampus (yellow region) and other brain regions. However, the diffusion rate of Gd-DTPA into the brain is decreased in the Aqp4^−/−^-sham group. Following SAH, Gd-DTPA is accumulated within the subarachnoid space and ventricles, and only a small amount of Gd-DTPA is diffused into the brain parenchyma, especially in Aqp4^−/−^-SAH animals **(A)**. In the sham group, the maximum mean concentration of Gd-DTPA in the brain emerges at 0.5 h after injection and is declined to the baseline level at 3 h. The mean concentrations of Gd-DTPA in the Aqp4^−/−^-sham, SAH, and Aqp4^−/−^-SAH groups are significantly lower than that of the sham group at 0.5 h and are markedly higher than that of the sham group at 3 h. **(B)** analysis of the Gd-DTPA concentration shown in **(A)**. *p < 0.05 vs. the sham group, ^#^*p* < 0.05 vs. the Aqp4^−/−^-sham group, ^&^*p* < 0.05 vs. the SAH group, *n* = 6.

In sham group animals, the Gd-DTPA injected into the cisterna magna was widely distributed in the subarachnoid space and ventricles and gradually flowed into the brain parenchyma. However, the diffusion speed of Gd-DTPA into the brain was markedly decreased in the Aqp4^−/−^-sham group. Following SAH, a large quantity of Gd-DTPA was accumulated into the cisterna magna and the ventricles, and only a small amount of Gd-DTPA diffused into the brain parenchyma, especially in Aqp4^−/−^-SAH animals (Figure 2A). Statistics on the average concentration of Gd-DTPA within the brain parenchyma is shown in Figure 2B.

**Figure 3:**
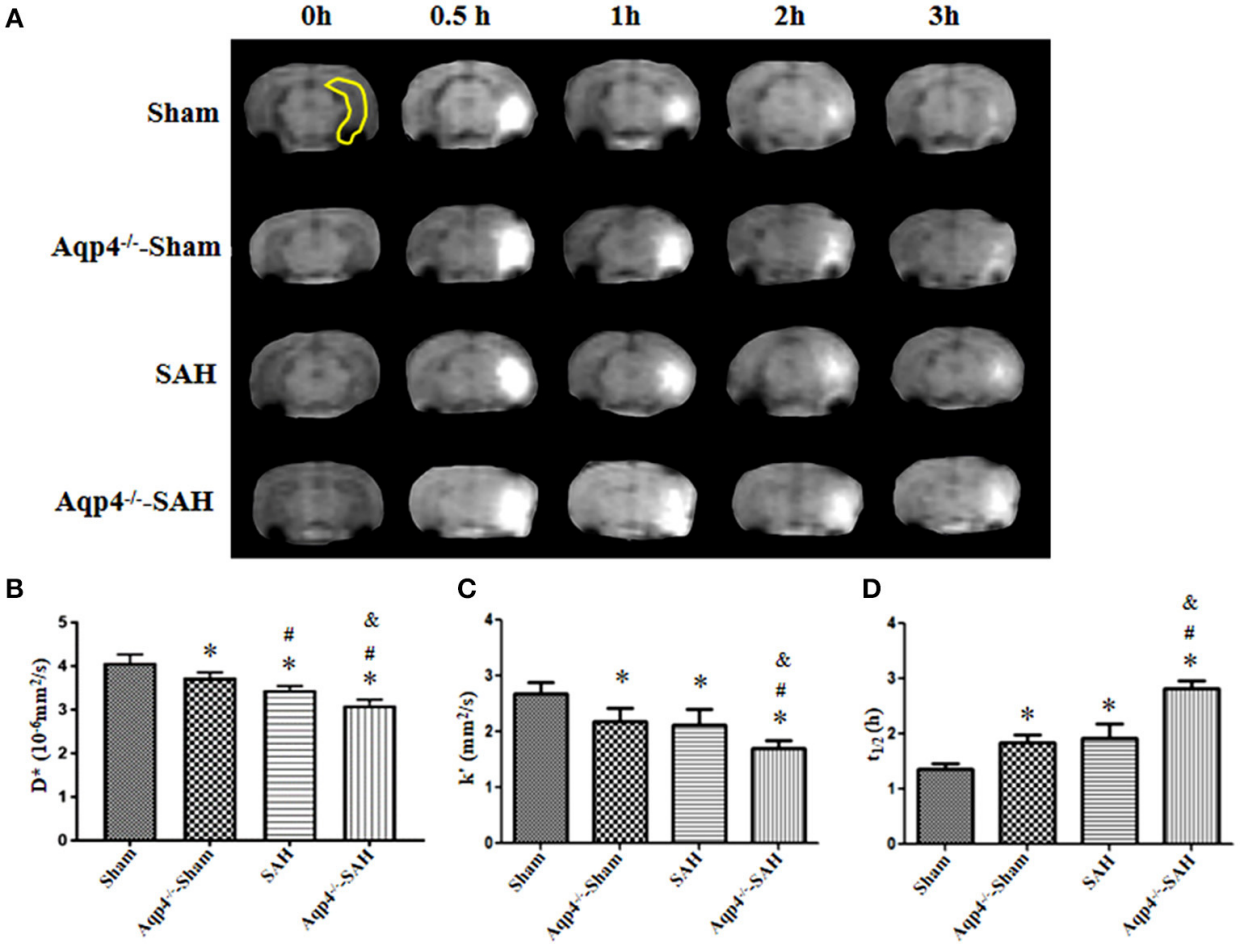
Distribution of Gd-DTPA injected into the hippocampus. In the sham group, Gd-DTPA within the hippocampus (yellow region) diffuses rapidly and is completely cleared at 3 h; the diffusion and clearance rates of Gd-DTPA in the Aqp4^−/−^-sham group are reduced, and a small amount of Gd-DTPA still remains in the hippocampus at 3 h; in both SAH and Aqp4^−/−^-SAH groups, the diffusion and clearance rates of Gd-DTPA in the hippocampus are significantly decreased, and abundant Gd-DTPA is maintained in the hippocampus at 3 h **(A)**. **(B–D)** show the analysis for the diffusion rate, D*; the clearance rate, k′; and the half-life, t_1/2_, of Gd-DTPA in the hippocampus of each group. *p < 0.05 vs. the sham group, ^#^*p* < 0.05 vs. the Aqp4^−/−^-sham group, ^&^*p* < 0.05 vs. the SAH group, *n* = 6.

Additionally, in the sham group, Gd-DTPA injected into the hippocampus diffused rapidly and cleared away at 3 h post injection, with the diffusion rate D^*^ = (3.96 ± 0.12) × 10^−6^ mm^2^/s, the clearance rate k′ = (2.55 ± 0.16) × 10^−4^/s, and the half-life t_1/2_ = (0.77 ± 0.17) h. However, the diffusion and clearance of Gd-DTPA in the Aqp4^−/−^-sham group was slightly slowed down, and a small amount of Gd-DTPA remained in the hippocampus at 3 h after injection, D^*^ = (3.36 ± 0.09) × 10^−6^ mm^2^/s, k′ = (2.22 ± 0.13) × 10^−4^/s, and t_1/2_ = (1.16 ± 0.07) h. In both the SAH and Aqp4^−/−^-SAH groups, the diffusion and clearance of Gd-DTPA in the hippocampus were significantly decreased, and there was a great number of Gd-DTPA in the hippocampus at 3 h post injection (Figure 3A). The D^*^, k′, and t_1/2_ were (2.93 ± 0.18) × 10^−6^ mm^2^/s, (1.84 ± 0.21) × 10^−4^/s, and (1.41 ± 0.11) h in the SAH group and were (2.18 ± 0.18) × 10^−6^ mm^2^/s, (1.29 ± 0.14) × 10^−4^/s, and (1.73 ± 0.25) h in the SAH-Aqp4^−/−^ group, respectively. There was a significant difference in D^*^, k′, and t_1/2_ among the four groups (*p* < 0.05, Figures 3B–D).

### Fluorescence Imaging

**Figure 4:**
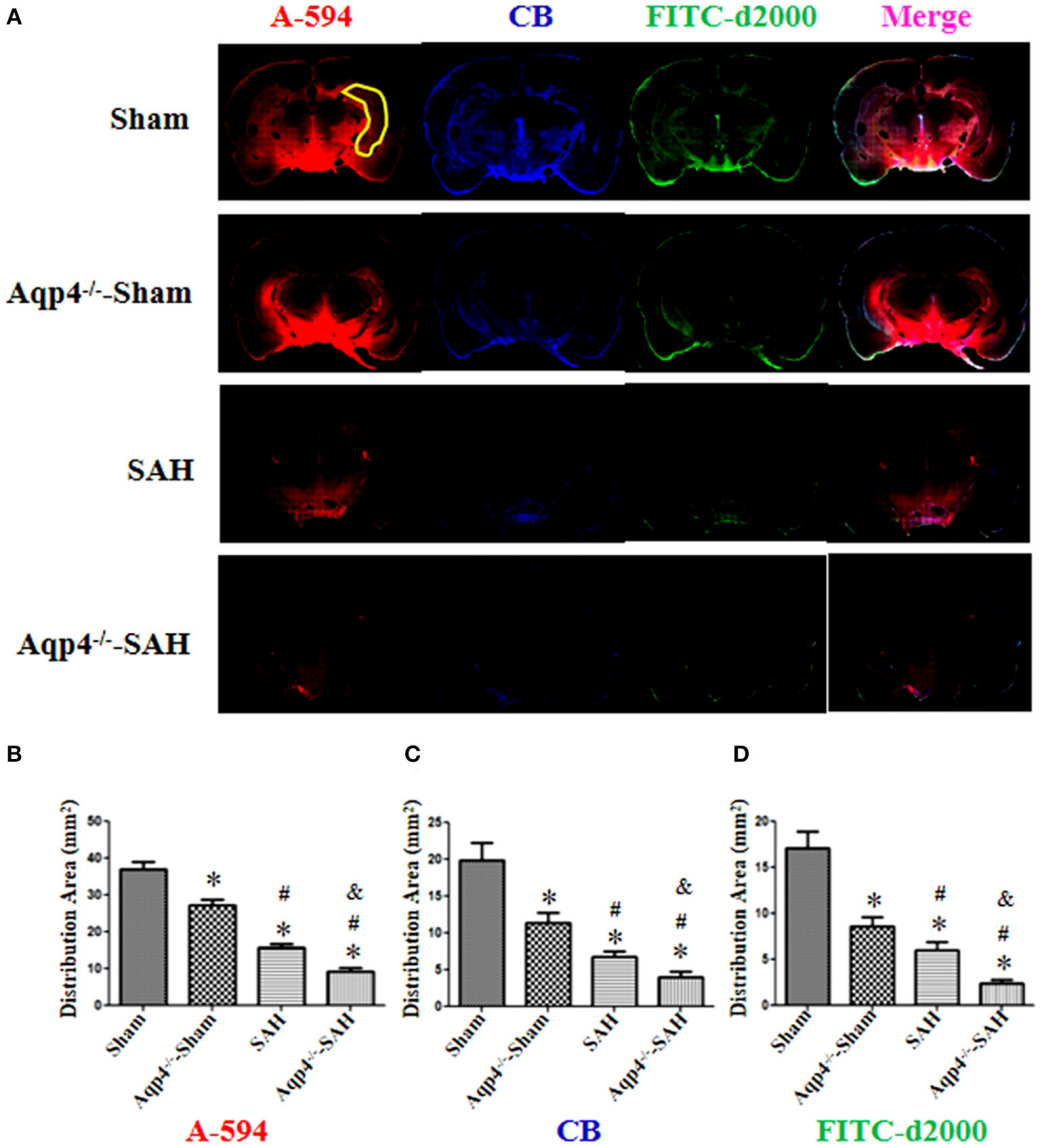
The distribution of fluorescence dyes injected into the cisterna magna. **(A)** In the sham group, the fluorescence mixture of A594 (micro-molecule), CB (middle-molecule), and FITC-d2000 (macro-molecule) injected into the cisterna magna is quickly distributed in the hippocampus (yellow region) at 0.5 h. The distribution area of A-594 is the largest compared with those of CB and FITC-d2000, and the distribution range of FITC-d2000 is the smallest. The distribution areas of each fluorescence dye in the Aqp4^−/−^-sham, SAH, and Aqp4^−/−^-SAH groups are significantly decreased compared with that of the sham group, especially in the Aqp4^−/−^-SAH group. **(B–D)** show the distribution area of fluorescence dyes of each group., *p < 0.05 vs. the sham group, ^#^*p* < 0.05 vs. the Aqp4^−/−^-sham group, ^&^*p* < 0.05 vs. the SAH group, *n* = 6.

As shown in Figure 4A, in the sham group, the fluorescence mixture of A594, CB, and FITC-d2000 injected into the cisterna magna diffused into the hippocampus quickly. The micro-molecule fluorescence dye A594 had the largest distribution area, followed by CB and FITC-d2000. Compared with the distribution area of A594, CB, and FITC-d2000 in the sham group, the areas of these fluorescence dyes in the Aqp4^−/−^-sham, SAH, and Aqp4^−/−^-SAH groups were significantly decreased (*p* < 0.05, Figure 4A). The analysis on the average area of these fluorescence dyes within the brain parenchyma is shown in Figures 4B–D.

**Figure 5:**
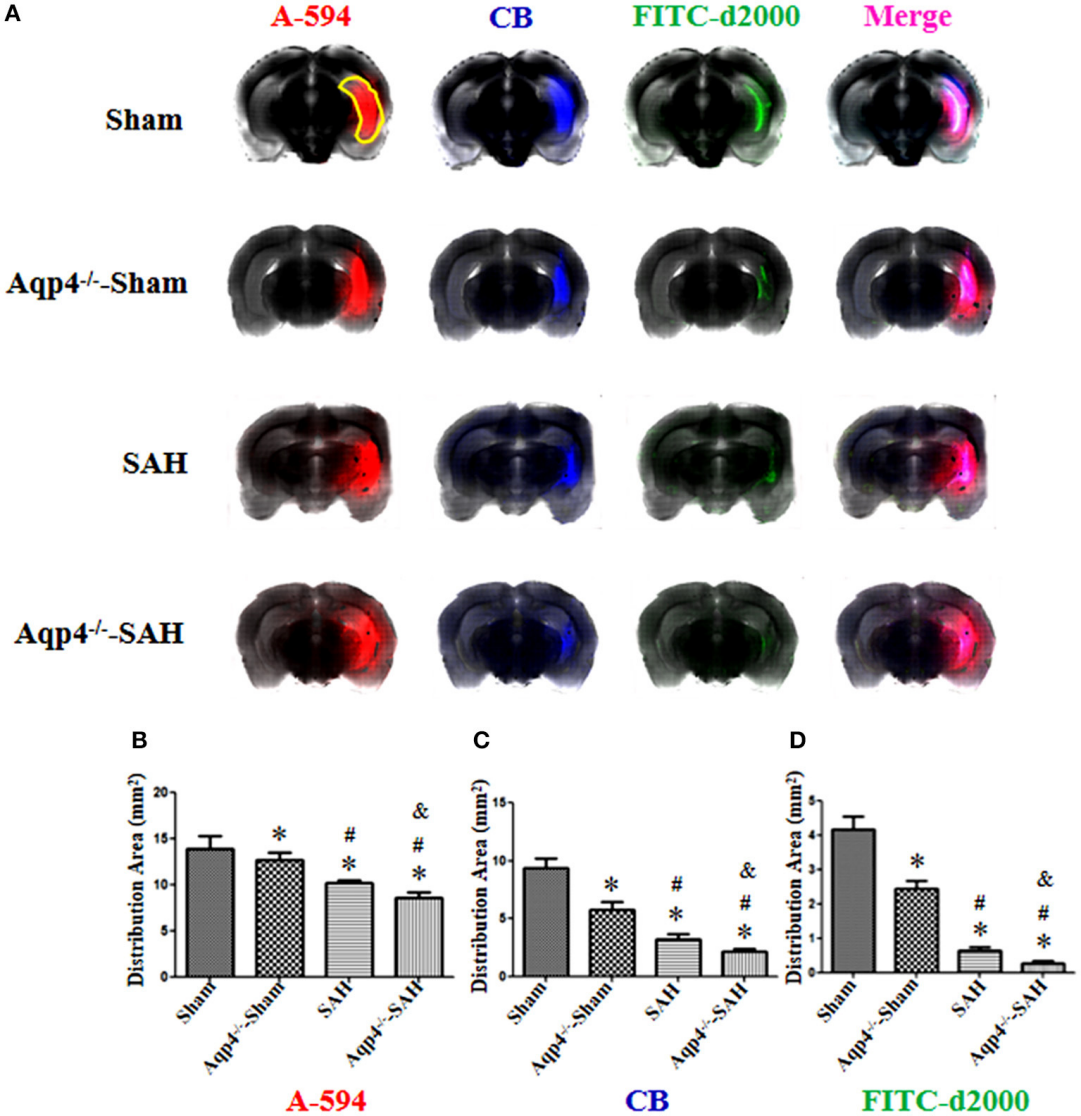
The distribution of fluorescence dyes injected into the hippocampus. **(A)** In the sham group, the fluorescence mixture of A594, CB, and FITC-d2000 injected into the hippocampus (yellow region) diffuses quickly within the hippocampus and adjacent brain parenchyma, the distribution area of A-594 is the largest compared with those of CB and FITC-d2000. The distribution areas of A594, CB, and FITC-d2000 in the Aqp4^−/−^-sham, SAH and Aqp4^−/−^-SAH groups are significantly decreased compared with that of the sham group, especially in the Aqp4^−/−^-SAH group. **(B–D)** show the distribution area of fluorescence dyes each group, *p < 0.05 vs. sham group, ^#^*p* < 0.05 vs. Aqp4^−/−^-sham group, ^&^*p* < 0.05 vs. SAH group, *n* = 6.

As shown in Figure 5, the fluorescence mixture of A594, CB, and FITC-d2000 injected into the hippocampus diffused into the adjacent brain parenchyma. Compared with the fluorescence distribution area in the sham group, the distribution areas of A594, CB, and FITC-d2000 in the Aqp4^−/−^-sham, SAH, and Aqp4^−/−^-SAH groups were significantly decreased, especially in the Aqp4^−/−^-SAH group (*p* < 0.05, Figures 5B–D).

### Transmission Electron Microscopy Examination

**Figure 6:**
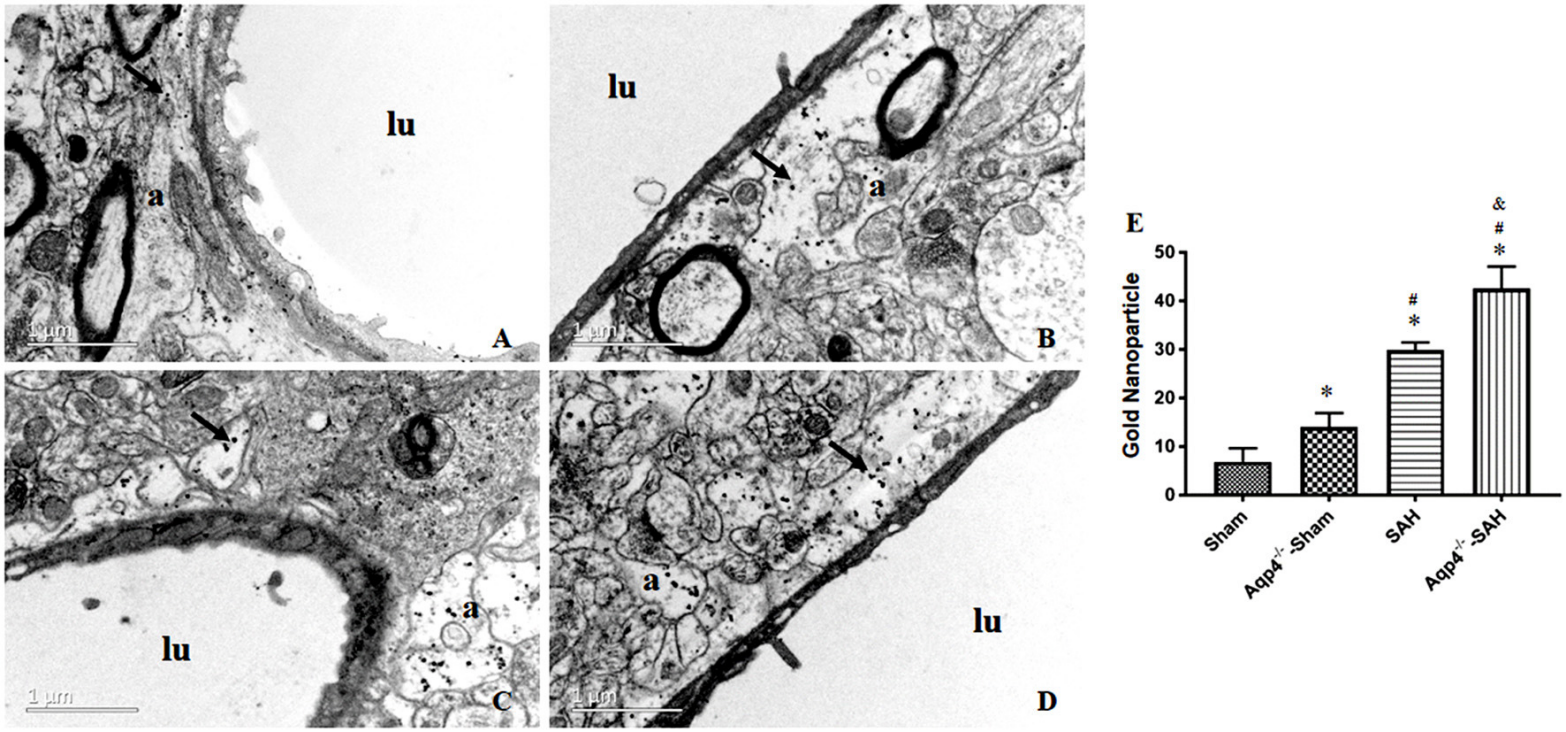
The distribution of gold nanoparticles in hippocampus perivascular space. In the sham group, the gold nanoparticles in perivascular space are almost cleared, and a few are engulfed by the surrounding astrocytes at 3 h after injection **(A)**. In the Aqp4^−/−^-sham group, there are markedly increased gold nanoparticles accumulation in the swelling astrocyte endfeet **(B)**. After SAH, numerous gold nanoparticles are maintained in astrocyte endfeet with obvious edema, especially in the Aqp4^−/−^-SAH group **(C,D)**. “lu” indicates capillary lumen, “a” indicates astrocyte. Scale bar = 1 μm. **(E)** shows the analysis for the number of gold nanoparticles in each group. *p < 0.05 vs. the sham group, ^#^*p* < 0.05 vs. the Aqp4^−/−^-sham group, ^&^*p* < 0.05 vs. the SAH group, *n* = 6.

In the sham group, most of the gold nanoparticles in the hippocampus were cleared, and only a few were engulfed by the perivascular astrocytes. In the Aqp4^−/−^-sham group, the astrocytes endfeet had mild edema, and a few gold nanoparticles were accumulated in the endfeet. After SAH, numerous gold nanoparticles were gathered in the astrocytes with obvious edema, especially in the Aqp4^−/−^-SAH group (Figure 6). We randomly selected ten electron microscope images and analyzed the number of gold nanoparticles. The statistical results are shown in Figure 6E.

### Immunofluorescence Staining

**Figure 7:**
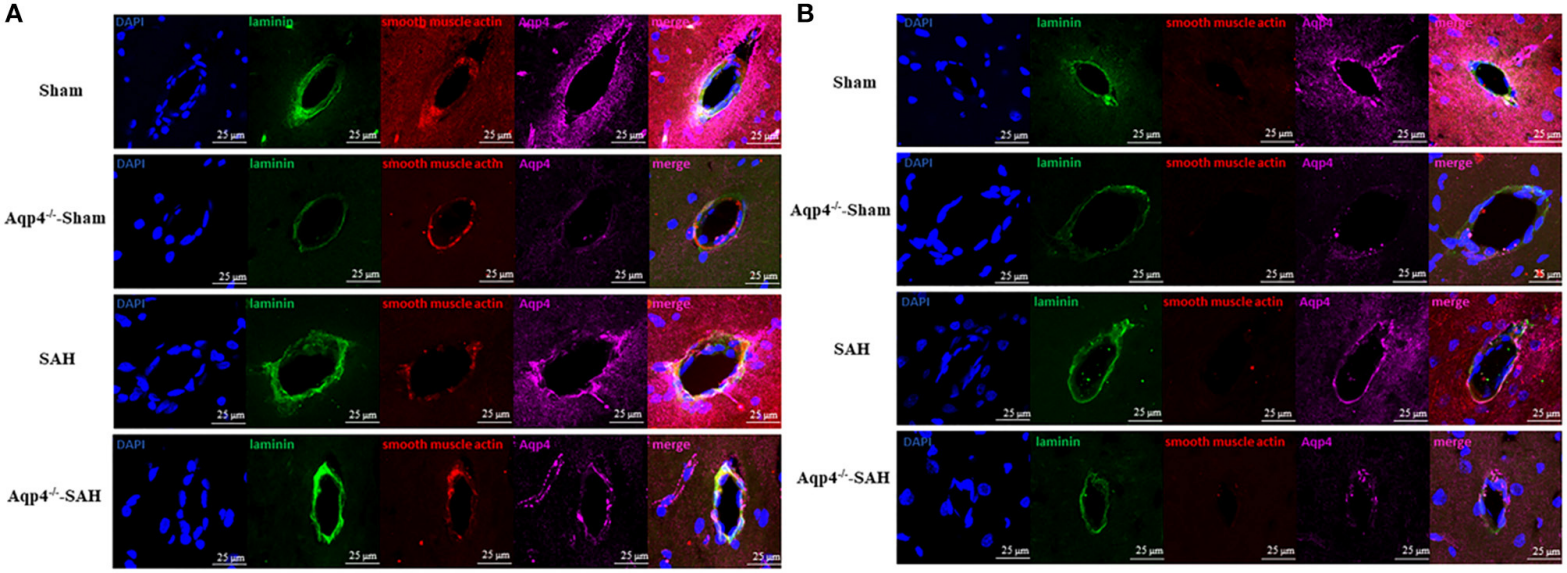
The expression of Aqp4 around the arteriole and the venule in the hippocampus. **(A)** shows the expression of Aqp4 around the arteriole (positive staining for both laminins and smooth muscle actin). In the sham group, the Aqp4 protein is evenly distributed around the arteriole. After SAH, the expression level of Aqp4 around the arteriole is markedly increased compared with that of the sham group. Rare Aqp4 is detected in the Aqp4^−/−^-sham and Aqp4^−/−^-SAH groups. **(B)** shows the expression of Aqp4 around the venule (positive staining for laminins but negative for smooth muscle actin). In the sham group, the expression level of Aqp4 around the venule is lower than that of the arteriole. In the SAH group, the expression level of Aqp4 around the venule does not change noticeably. Rare Aqp4 is detected in the Aqp4^−/−^-sham and Aqp4^−/−^-SAH groups. Scale bars = 25 μm.

The immunofluorescence staining results indicated that, after SAH, the expression level of Aqp4 protein around the arteries was markedly increased compared with that of the sham group. Meanwhile, the expression level of Aqp4 around the arteries was significantly higher than that around the veins following SAH. Rare Aqp4 protein was detected in both Aqp4^−/−^-sham and Aqp4^−/−^-SAH groups (Figures 7A,B).

## Discussion

In this study, the results of MRI, fluorescence imaging, and TEM examination revealed that the GS in the brain was impaired after SAH, which could be aggravated after Aqp4 deletion.

The GS is located at the PAS (i.e., Virchow-Robin space) surrounding the vasculature system in the brain (Iliff et al., 2012; Xie et al., 2013). The GS can facilitate the entrance of the CSF into the brain parenchyma and the clearance of ISF from the brain mediated by the astrocytes (Iliff et al., 2012, 2013).

As the most abundant water channel in the brain, Aqp4 is highly expressed at the perivascular astrocyte endfeet (Iliff et al., 2012; Kress et al., 2014). The Aqp4 protein mediates the influx of CSF into the brain parenchyma along the PAS and the efflux of ISF along the perivascular space into the subarachnoid space (Tarasoff-Conway et al., 2015; Plog and Nedergaard, 2018). In this study, we found that the inflow and outflow of ISF tracers (fluorescence tracer and Gd-DTPA tracer) in the brain were noticeably decreased after Aqp4 deletion. Furthermore, there were more gold nanoparticles accumulated in the interstitial space in the Aqp4^−/−^-sham group than that of the sham group, which might be due to GS dysfunction following Aqp4 knockout.

It was reported that the GS was damaged during the acute phase of SAH and ischemic stroke (Sun et al., 2006, 2011), which consequently led to the accumulation of metabolites and toxic factors (de Rooij et al., 2013). Furthermore, the GS dysfunction aggravates the delayed vasospasm or microcirculatory impairment (Siler et al., 2014). Therefore, restoring the normal GS function might be a promising strategy to improve the outcome of SAH patients.

In this study, the expression level of Aqp4 was highly increased in the arteries after SAH; meanwhile, the level of Aqp4 in the veins did not change markedly. The previous studies indicated that the astrocytes were activated to enhance the expression of Aqp4 following SAH (Chen et al., 2017; El Amki et al., 2018). After SAH, the extravasated blood constituents produced ischemia and sterile inflammation (Lucke-Wold et al., 2016; Schneider et al., 2018) that could activate the astrocytes surrounding the arteries and increase the expression level of the Aqp4 protein, which was slightly expressed on the venous site. Therefore, we speculated that the inflow of CSF from PAS was highly increased due to the enhanced level of Aqp4, and the ISF volume was expanded owing to the unchanged expression of Aqp4 in the veins.

By using the TEM method, we found that the clearance rate of gold nanoparticles in the interstitial space was significantly decreased after SAH, especially in the Aqp4^−/−^-SAH group. These results implied that there was an obvious GS impairment after SAH; therefore, the toxic factors (such as apoptotic and inflammatory mediators) accumulating in the brain parenchyma could lead to the dysfunction of the neurovascular units and consequently aggravated the neurological deficits after SAH, as the results showed in Figure 1A. Therefore, it seemed that preserving the Aqp4 expression level might be an effective method to maintain the GS function in various neurological diseases (Kress et al., 2014; Yin et al., 2018).

MRI (T1WI) method was applied to detect the CSF–ISF flow in the GS by using Gd-DTPA contrast in this study. Due to its impermeability to the cellular membrane, Gd-DTPA has been proved an ideal indicator for observing CSF–ISF flow (Han et al., 2014). Compared with other methods, such as the tetramethylammonium (TMA^+^) method and the integrative optical imaging (IOI) method (Nicholson and Phillips, 1981; Nicholson, 1992; Xiao et al., 2008), the MRI method can explore the ISF drainage in the deep brain region (e.g., the hippocampus in this study) with a three-dimensional model. Nowadays, the MRI method has been widely used to explore the mechanism of some brain diseases (Ten Kate et al., 2018).

In this study, the hippocampus was chosen as the region of interest because it is particularly susceptible to ischemia (Stoltenburg-Didinger, 1994). In addition, we have used the male rats to explore the ISF drainage system injury after SAH. It is well-known that the estrogen in female animals can play significant protective roles after SAH (Ding et al., 2014), and there will be many complicated factors to interfere with the results if we used both male and female rats in this study. In the future study, we will perform a similar study by using female animals to explore the sex differences in the GS impairment and the roles of Aqp4 after SAH.

The brain water content evaluation method we used in this study could assess that the net increase of water resulted from vasogenic edema in the brain, which was independent of the Aqp4 function and occurred through the intercellular spaces of the endothelial cells in the microvessels. That is, Aqp4 can just modulate the CSF inflow into the brain (or ISF outflow from the brain) through the perivascular space other than the endothelial intercellular spaces. Therefore, Aqp4 knockout in the sham animal does not influence the brain water content (due to healthy intercellular spaces of the endothelial cells). In the same way, after SAH, as we showed in this study, BBB disruption and intercellular junction injury could lead to obvious brain edema and increase brain water content, regardless of the Aqp4 expression level.

In summary, this study revealed that the inflow of CSF into the brain and outflow of ISF through the GS were markedly blocked after SAH, especially in the Aqp4^−/−^ rats. Additionally, based on the effects of Aqp4 knockout on brain edema development and neurological deficits after SAH, we inferred that Aqp4 could not only alleviate brain edema as reported previously (Blixt et al., 2018) but also facilitate the flow between CSF and ISF to eliminate the toxic factors in the GS and attenuate the neutrocyte loss and BBB disruption. Therefore, Aqp4 knockout led to toxic substance accumulation in the brain and prolonged injury to the neutrocytes and BBB after SAH.

## Data Availability Statement

The original contributions presented in the study are included in the article/supplementary material, further inquiries can be directed to the corresponding author/s.

## Ethics Statement

The animal study was reviewed and approved by the Ethics Committee of Peking University Health Center.

## Author Contributions

EL, YaZ, and XY performed the immunohistochemistry staining. EL, YiZ, and LS performed the Western blot. XP and HM analyzed the data and refined the manuscript. JY conceived and coordinated the study. All authors contributed to the article and approved the submitted version.

## Conflict of Interest

The authors declare that the research was conducted in the absence of any commercial or financial relationships that could be construed as a potential conflict of interest.
